# Bacillus Calmette-Guérin Vaccine-Related Osteomyelitis in Immunocompetent Children in Saudi Arabia: A Narrative Review

**DOI:** 10.7759/cureus.32762

**Published:** 2022-12-21

**Authors:** Abdulsalam Alawfi

**Affiliations:** 1 Pediatric Infectious Diseases, Taibah University, Madinah, SAU

**Keywords:** antituberculous interventions, immunocompetent, osteomyelitis, children, bacillus calmette-guérin

## Abstract

The Bacillus Calmette-Guérin (BCG) vaccine is the most frequently used live-attenuated vaccine worldwide. Since 2002, two BCG vaccination strains, Pasteur 1173 P2 and Tokyo 172-1, have been the mainstay of Saudi Arabian healthcare. In 2005, the Danish 1331 strain was first used as the principal strain in clinical trials. Children can develop osteomyelitis 4-24 months after immunization with the BCG vaccine, an uncommon but serious side effect in immunocompetent children. We conducted this study to review the epidemiology, diagnosis, clinical symptoms, laboratory analyses, imaging features, and management of BCG osteomyelitis in immunocompetent children. Long bone metaphyses and epiphyses are more frequently affected. The diagnosis of BCG osteomyelitis is difficult because the symptoms are vague and subtle, and the duration between presentation and vaccination may range from a few months to a year. Radiography and computed tomography scans for BCG osteomyelitis typically show a devastating lesion with an associated periosteal response. Magnetic resonance imaging frequently reveals a large interosseous abscess indicative of osteomyelitis. There are no current treatment guidelines for BCG osteomyelitis in Saudi Arabia, but antituberculous regimens, particularly isoniazid and rifampicin, have been found to be very effective in previous studies. Although older studies did not favor surgical intervention because of the risk of complications, a few studies performed minor surgical interventions and had good outcomes. As BCG osteomyelitis is an infrequent complication, especially in immunocompetent children, its diagnosis is time-consuming. Therefore, it is critical to inform healthcare workers of this possible complication to make the diagnosis more straightforward and avoid confusion with pyogenic osteomyelitis. As only a few cases have been reported, further studies in Saudi Arabia are required for evidence-based guidelines applicable to actual practice to be established.

## Introduction and background

The Bacillus Calmette-Guérin (BCG) vaccine is a live attenuated immunization administered worldwide. Since Calmette and Guérin first developed the vaccine in 1921, around three billion people have been given the opportunity to protect themselves against the disease by receiving vaccinations [1]. The BCG vaccine helps prevent tuberculosis and is especially useful in treating active sicknesses, such as tuberculous (TB) meningitis and disseminated tuberculosis in children [2].

Currently, there is no mechanism for evaluating which of the already existing strains of BCG vaccination is the most efficient at eliciting a protective immune response. As a result, there is no general agreement on which strain is the most suitable for broad coverage. Many different strains are used worldwide. Each year, the United Nations Children's Fund (UNICEF) provides more than 120 million doses of the BCG vaccination to recipients in more than 100 countries [3]. The BCG-Denmark, BCG-Japan, and BCG-Bulgaria strains developed by UNICEF are the most commonly used strains worldwide (Supply Division of UNICEF, personal communication) [3]. Some underdeveloped countries are currently using multiple strains of the BCG vaccine simultaneously for vaccination purposes. In contrast, the use of only a single strain is often permitted in most developed nations. Despite this, a few countries produce and sell their own versions of the BCG vaccination [4].

The Pasteur 1173 P2 and Tokyo 172-1 strains are the most frequently used BCG vaccines in Saudi Arabia. In 2005, the Danish 1331 strain was designated as the most advantageous strain of choice for use for the first time [5]. Following the vaccine adjustment in 2006, the incidence of infants with BCG-related complications was higher than previously observed, at a rate of 3.12 to 10.14 complications/1,000 newborns. BCG lymphadenopathy was the most common complication [5,6].

According to several studies, the BCG vaccine puts children at an increased risk of developing osteomyelitis and infectious arthritis [7,8]. These findings apply to children who have sound immune systems as well as those who have immunological deficits [7]. BCG can spread rapidly throughout the body if it is placed in or just under the skin. Within an hour of the delivery of microorganisms, they are discovered in various organs. Children can acquire osteomyelitis 4-24 months after receiving the BCG vaccine. Osteomyelitis is a rare, but significant adverse effect of BCG vaccination in immunocompetent hosts. It is an infectious condition that causes bone destruction, with significant pain and inflammation [8].

Osteomyelitis caused by BCG infection can manifest in one of the following two ways: locally at the site of the injection or throughout the body due to the spread of BCG infection. Metaphyseal and epiphyseal lesions of long bones are rather prevalent [9]. Following vaccination, the incubation period for BCG osteomyelitis is typically >6 months, and the long bones are most frequently affected, especially their metaphysis and epiphysis. On the other hand, osteomyelitis caused by Mycobacterium tuberculosis is more common in the spine and weight-bearing joints of older children and adults [9]. According to various authors, the onset of symptoms of BCG osteomyelitis may occur at a variable interval after BCG vaccination, ranging from three months to five years [7,9,10]. Owing to the link of the age of administration with BCG-associated complications, as suggested by some authors, Saudi Arabia and other countries changed the administration period from birth to six months of age [11,12]. However, a cohort study by Yang et al. [12] did not find any advantage to delaying BCG vaccine administration to six months instead of administering it at birth. We conducted this study to further review the epidemiology, diagnosis, clinical symptoms, laboratory analyses, imaging features, and management of BCG osteomyelitis in immunocompetent children.

## Review

Epidemiology

In a study conducted in Japan using a multi-puncture method, the incidence of BCG osteomyelitis was reported to be 0.01 per million vaccines. In contrast, a study conducted in Finland using the same methodology revealed that the incidence of BCG osteomyelitis was 30 per million (intradermal technique) [9,13]. Saudi Arabian infants are vaccinated with BCG within the first year of life; however, the rare incidence of BCG osteomyelitis in immunocompetent children has only been documented in a few studies [14]. Al-Arabi et al. [15] reported in 1984 that an immunocompetent child had presented at King Khaled University Hospital with pain and swelling around the left shoulder, with a history of BCG vaccination. In addition, in 2012, Al-Jassir et al. [14] from King Saud University reported three cases of culture-proven osteomyelitis that were confirmed by real-time polymerase chain reaction. In 2016, Alzomor et al. [16] reported a case that revealed osteomyelitis features in magnetic resonance imaging (MRI) with PCR positivity for the Mycobacterium bovis strain of BCG.

Diagnosis

The diagnosis of BCG-induced osteomyelitis is difficult; the symptoms are vague and subtle. The duration between presentation and vaccination may be a few months to a year [17]. The majority of studies have systematically evaluated the use of clinical, laboratory, PCR, culture, and radiology results for making the diagnosis.

Clinical presentation

The immunocompetent patients present with painless and palpable lymph nodes and laboratory data suggested either normal or significantly increased acute-phase reactants, according to Tsujioka et al. [17-20]. Those who were part of the BCG cohort investigated in this study had an average age of onset of 15.5 months and it took an average of 4.5 months to reach the final diagnosis [17]. Tsujioka et al. [17] found that among immunocompetent individuals, there were three cases of the sternum, two cases of humerus, two cases of the femur, and one case of proximal phalanx infection. In addition, Huang et al. [21] revealed that lower limbs were more affected by BCG osteomyelitis than other parts of the body, representing 60.6% of their patients. They also reported the percentage of each bone that was affected: lower long bones, 36.6%, foot, 23.9%; ribs or sternum, 15.5%; upper long bones, 9.9%, hands, 7%; and spine, 2.8%.

In Saudi Arabia, Al-Arabi et al. [15] described a 22-month-old child who presented with pain and two inches of swelling in the left arm and restricted shoulder mobility in all directions with no systemic disease; the only significant finding in the patient history was the administration of the BCG vaccine in the same arm when the child was 15 days old.

Al-Jassir et al. [14] from King Saud University reported three cases of BCG osteomyelitis. The first was an infant of 11 months who had been vaccinated against BCG seven months prior to presentation. The patient's right upper limb range of motion was reduced throughout the examination, and there was no history of trauma, fever, or other systemic symptoms. The other two patients also showed tenderness and complete knee movement limitation. The patients had received the BCG vaccine 10 and six months prior to presentation. Two patients suffered from limping and knee swelling: one on the right and the other on the left, with no history of trauma or systemic symptoms. In addition, Alzomor et al. [16] described a case of a seven-month-old child with no other systemic signs other than an asymptomatic swelling on the medial side of his foot for a duration of two weeks.

Clinical manifestations of BCG osteomyelitis are subtle and may only present with a mass that may or may not be painful, decreased range of movement, and limping. The majority of the literature did not report any systemic manifestations associated with the disease and patients presented within a few months after vaccination. The inconsistency of symptoms, and the infrequency of the disease, make the diagnosis difficult and may lead to delays in appropriate management.

Laboratory reports

Laboratories are critical for diagnosing BCG osteomyelitis by histological confirmation through culture or PCR. Mycobacterium culture may take nearly three weeks, and histopathology may reach an equivocal diagnosis; therefore, PCR is widely used in these cases. PCR can detect different strains of Mycobacterium, and recently, multiplex PCR has high capability to distinguish the BCG strain from the M. tuberculosis complex [17]. Acute phase reactants and white blood cells (WBC) may show a normal range or slight elevation [17].

Histological examination may be ineffective in diagnosis unless acid-fast bacillus staining is conducted [17-20]; however, previous studies have shown that acute-phase reactants and WBCs may show a normal range or only a slight elevation. The complete blood count and erythrocyte sedimentation rate (ESR) results from Alzomor et al. [16] indicated the average values. However, a sample from the right first metatarsal bone revealed granulomatous inflammation with caseation necrosis. A positive result for M. bovis, the most likely BCG strain, was obtained after three weeks of culture from tissue tested using acid-fast bacilli and PCR. In the study by Al-Jassir et al. [14], blood workup was normal, except for a minor increase in ESR. However, their bone sample showed caseating granulomatous inflammatory tissue, characteristic of TB osteomyelitis. Later, tissue PCR confirmed the presence of BCG osteomyelitis. Al-Arabi et al. [15] also found a slight elevation in ESR; when they explored the swelling, they found a granulomatous lesion with caseation, but direct microscopy and culture for M. tuberculosis and other bacteria were negative. Histopathology showed granulation tissue with many tuberculoid granulomas. It should be noted that this study was performed in 1984, and PCR was invented in 1983; therefore, PCR was not yet available in Saudi Arabia at that time [22].

Imaging

Imaging is a cornerstone to establishing a diagnosis of osteomyelitis. Tsujioka et al. [17] differentiated between pyogenic and BCG osteomyelitis using different imaging modalities to make it easier to consider BCG osteomyelitis. Additional imaging findings in the literature are discussed in the following sections.

Computed Tomography (CT) and Radiographs

**Figure 1:**
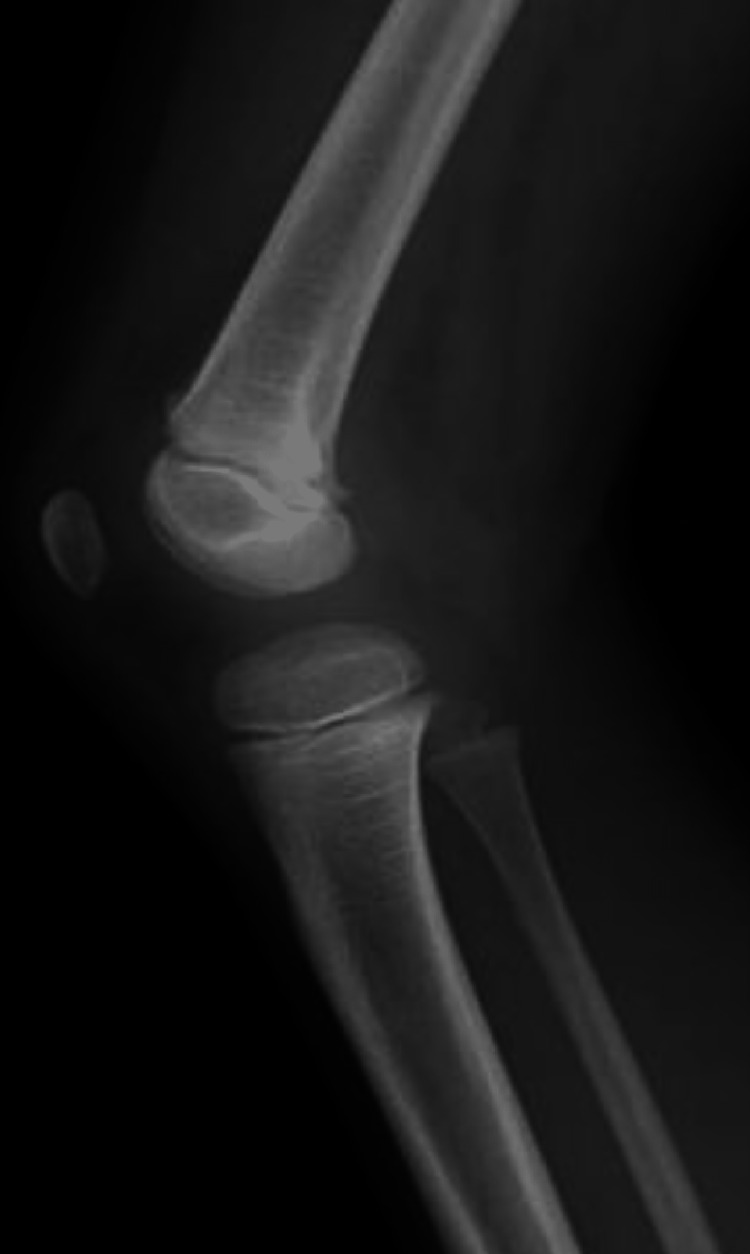
A six-year-old boy with staphylococcal osteomyelitis The lateral plain radiograph shows no obviously abnormal findings [17].

**Figure 2:**
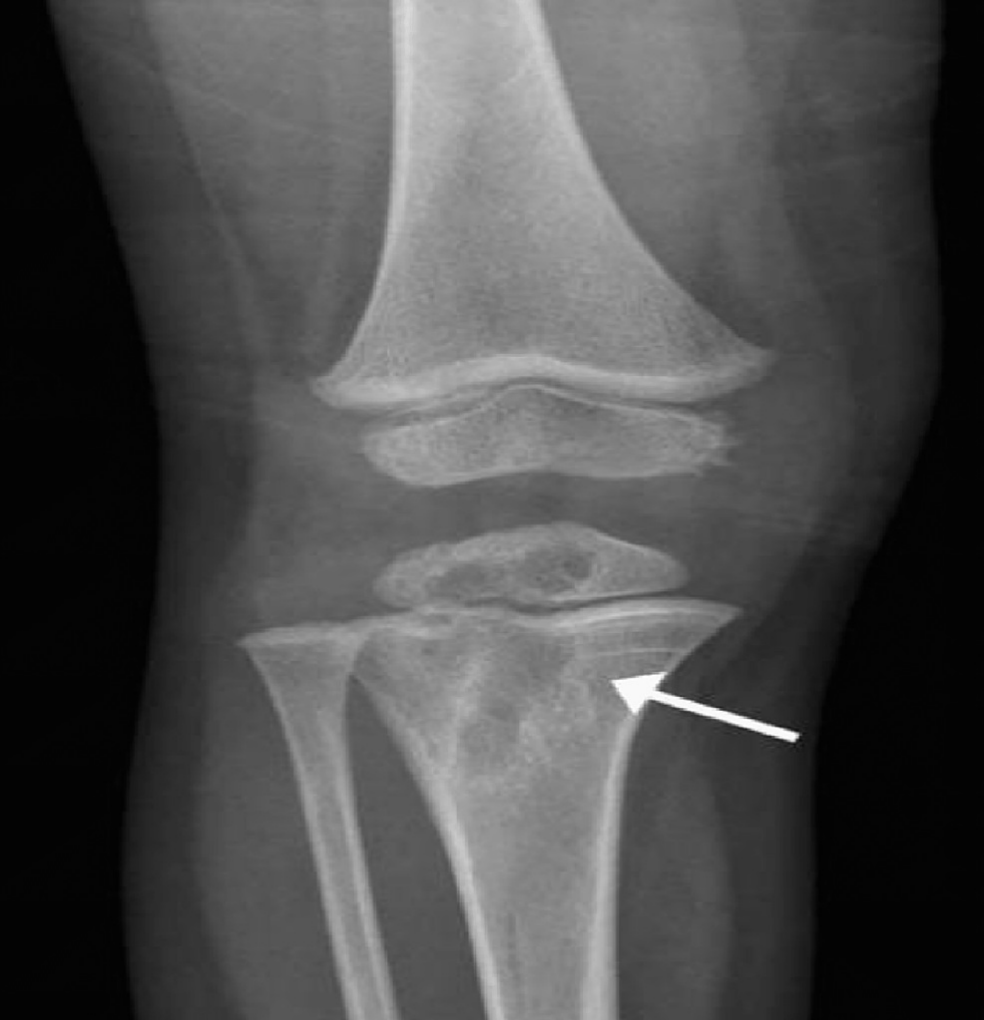
A 22-month-old girl with BCG osteomyelitis A plain radiograph shows an osteolytic lesion with well circumscribed sclerotic margin extending from the metaphysis, across the physis, and into the epiphysis [17].

**Figure 3:**
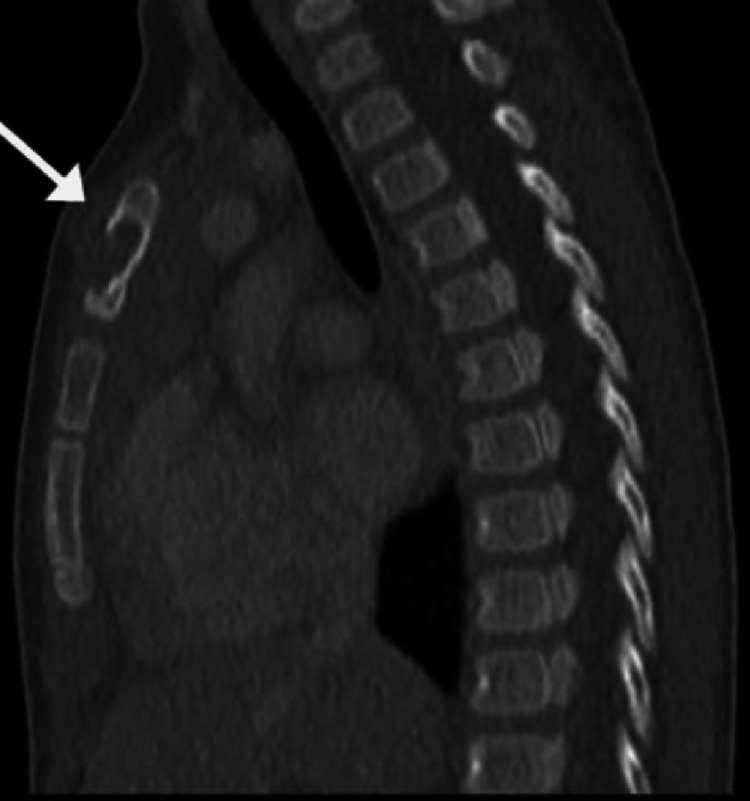
A 15-month-old girl with BCG osteomyelitis Sagittal reconstructed CT image shows a well-defined osteolytic lesion in the sternum with interruption of the anterior cortical continuity [17].

**Figure 4:**
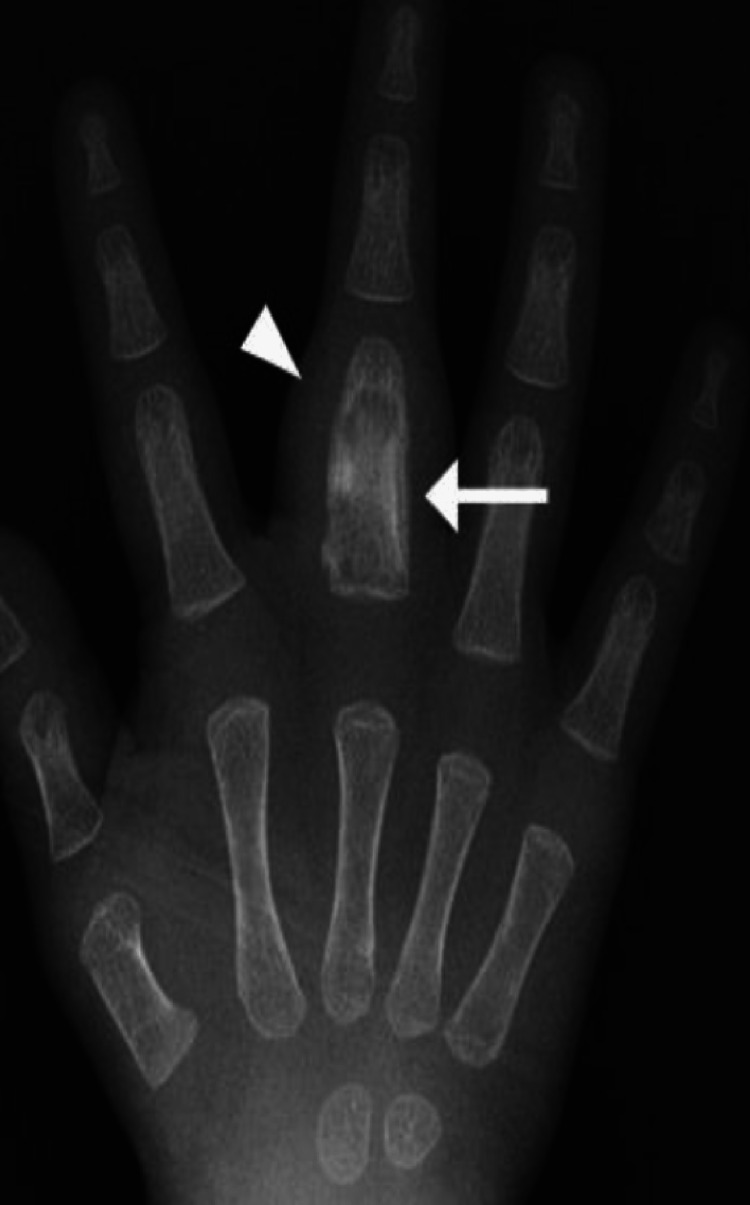
A 17-month-old girl diagnosed with BCG osteomyelitis of the phalanx The radiograph of the right hand shows destructive change and fusiform expansion of the proximal phalanx of the middle finger, which is depicted as multiple lytic lesions in the marrow and diaphyseal expansion (arrow). Noted is a single layer periosteal reaction (arrowhead) and prominent soft tissue swelling [17].

Tsujioka et al. [17] reported that 1) the initial plain radiograph frequently showed bone destruction in patients with BCG osteomyelitis; 2) intraosseous osteolytic lesions on plain radiography or CT and 3) periosteal reactions were more common in patients with BCG osteomyelitis than in those with pyogenic osteomyelitis (Figures 1-4).

**Figure 5:**
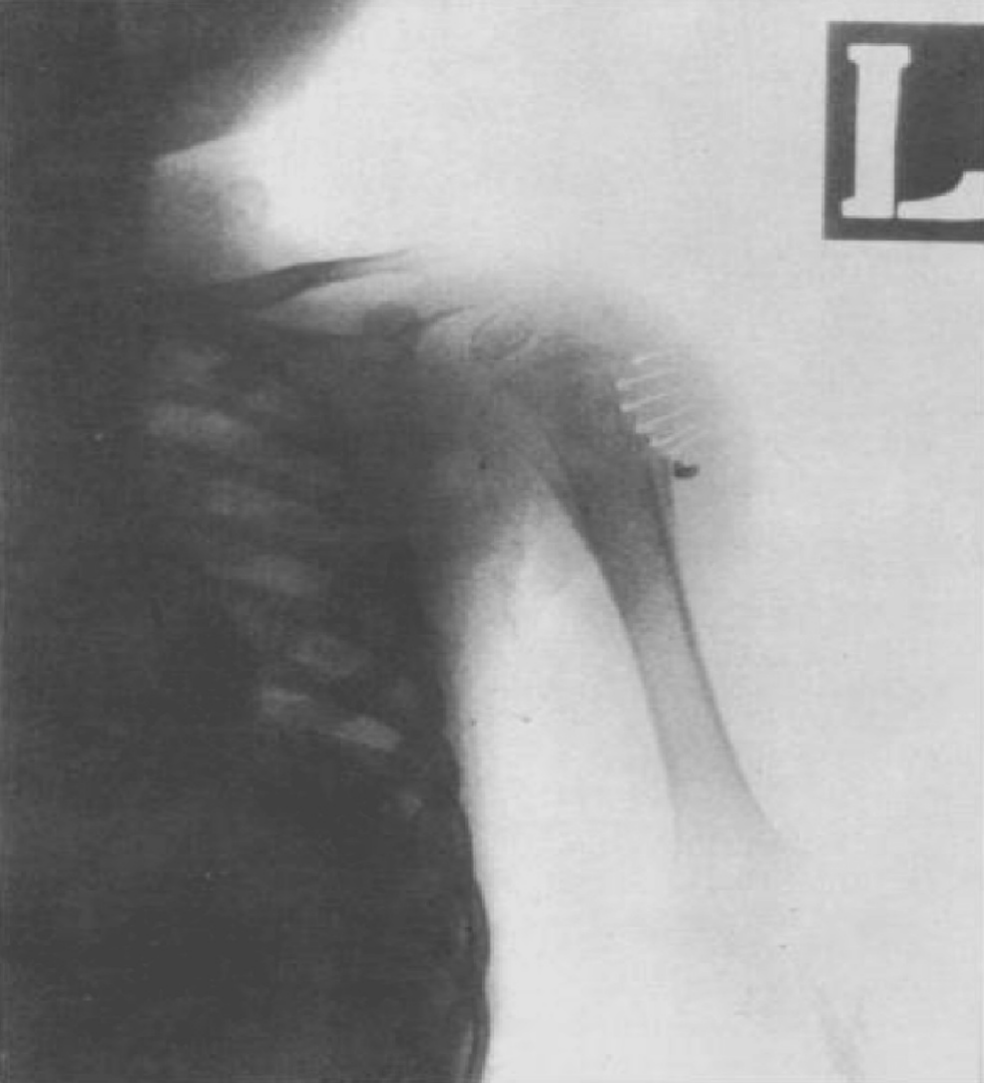
Plain radiograph showing destructive lesions in the left humeral metaphyseal area with an elevation of the periosteum and new bone formation

**Figure 6:**
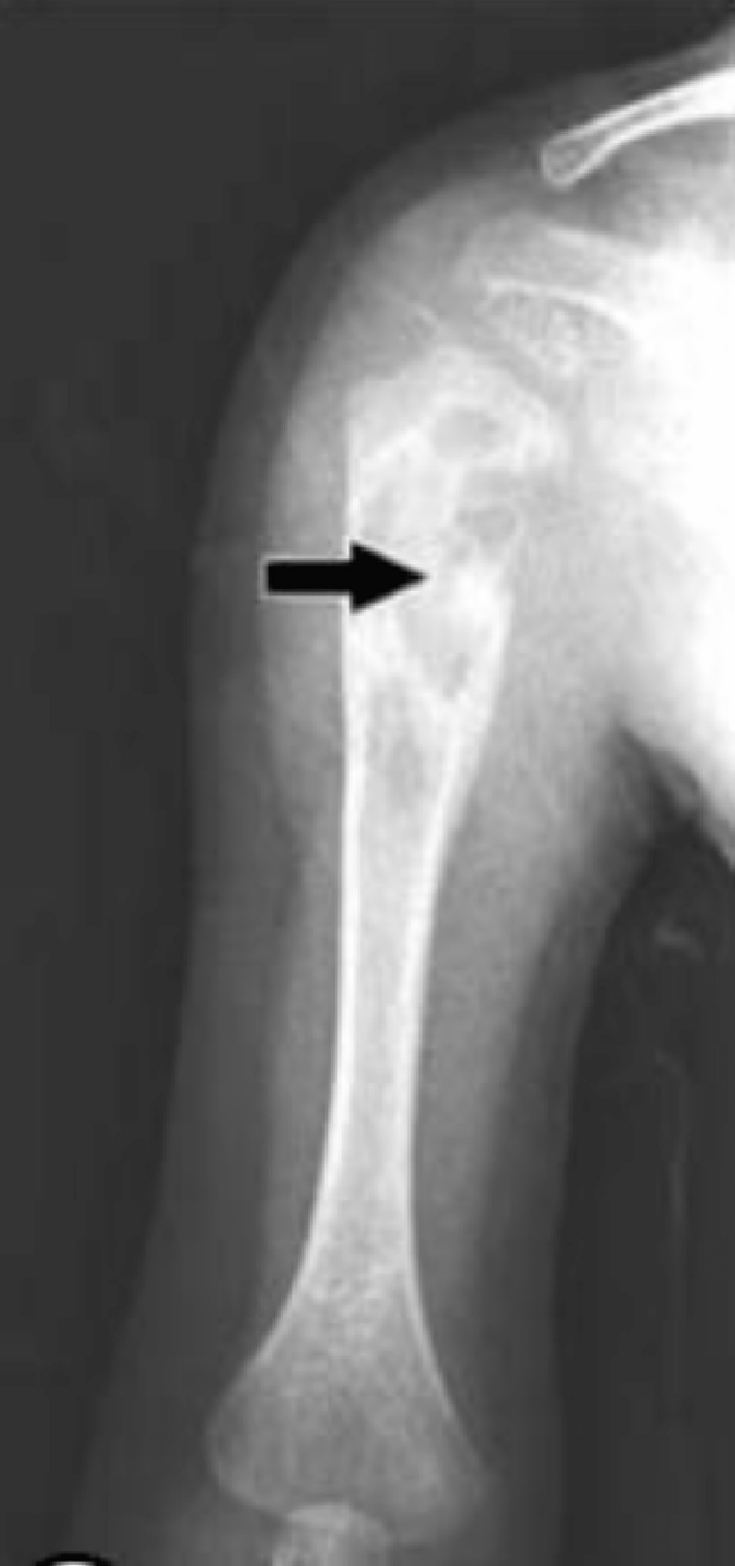
Plain radiograph showing destructive lesion upper metaphysis of the right humerus

**Figure 7:**
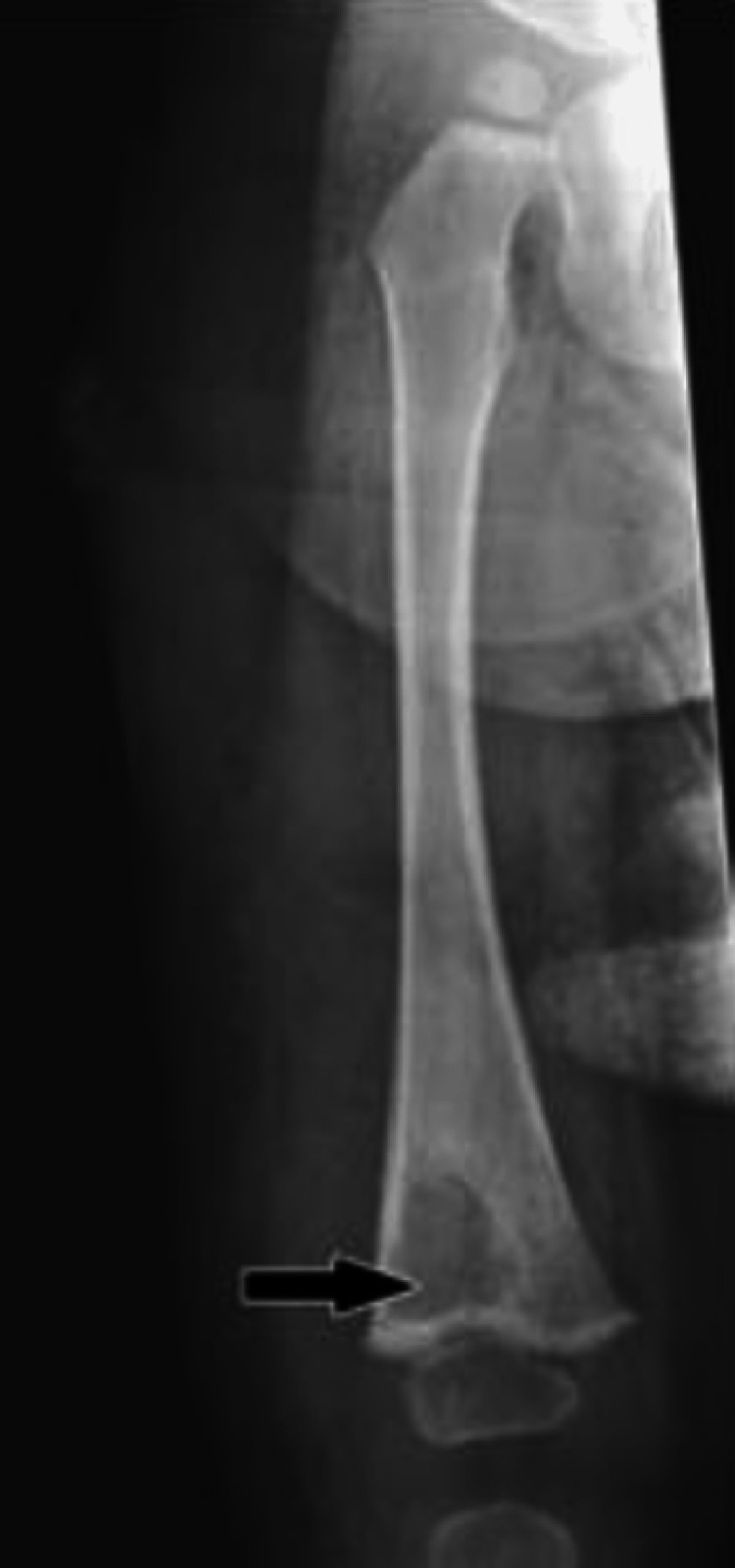
Radiological imaging showed a lytic lesion in the right distal femur with extensive soft tissue swelling

**Figure 8:**
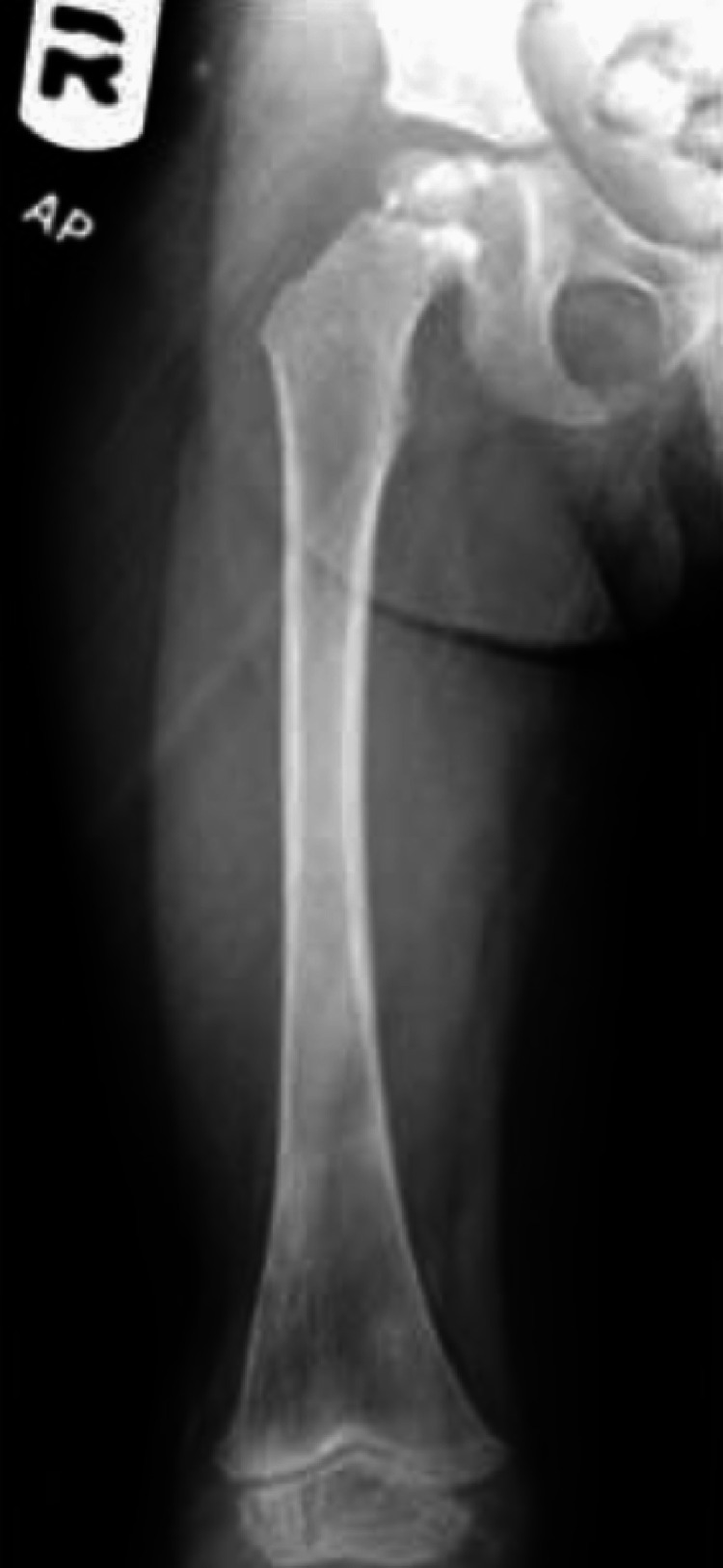
Final follow-up radiograph views showing complete resolution of the lesion

**Figure 9:**
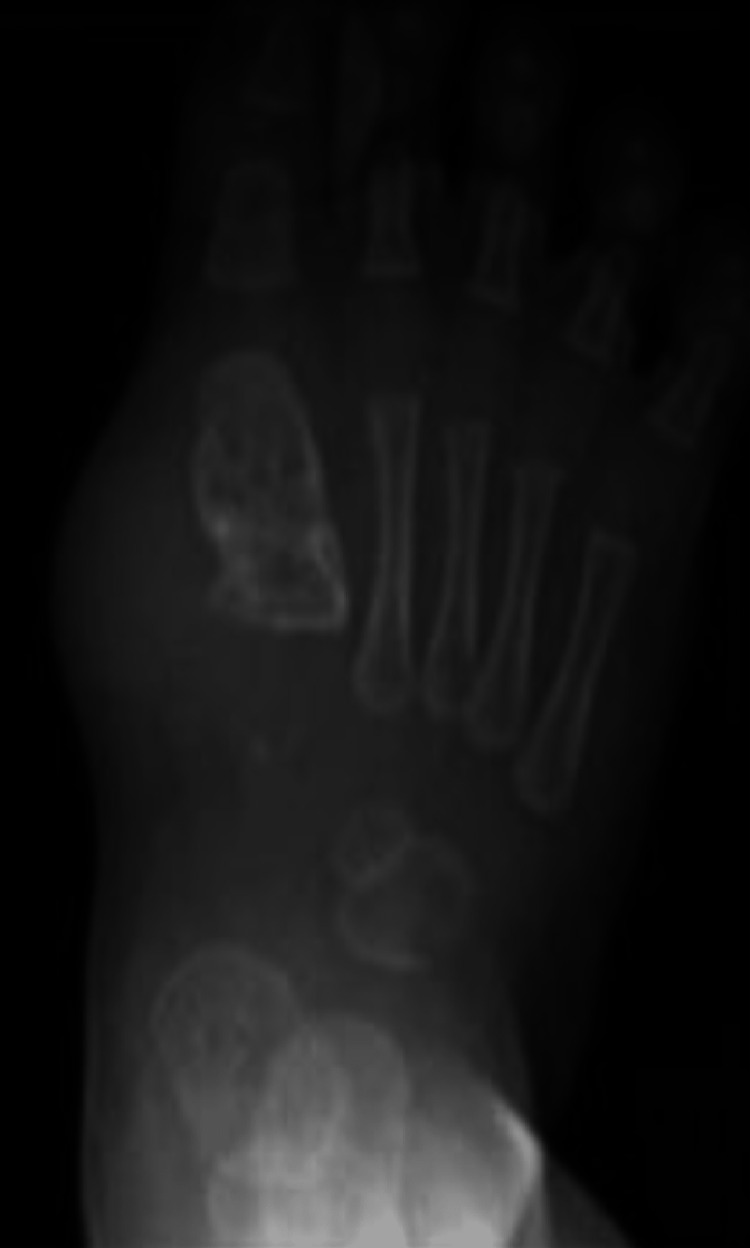
Plain radiograph of the right foot showing soft tissue swelling over the medial aspect of the right foot with multiple lytic areas in the first metatarsal bone with cortical breakthrough

In Saudi Arabia, Al-Arabi et al. [15] performed a radiographic analysis of their case. They found destructive lesions in the humeral metaphyseal area with an elevation of the periosteum and new bone formation (Figure 5). A plain radiograph in a study by Al-Jassir et al. [14] revealed a destructive lesion with cortical disruption (Figures 6-8). In addition, Alzomor et al. [16] found soft tissue swelling over the medial aspect of the right foot with multiple lytic areas in the first metatarsal bone and cortical breakthrough (Figure 9).

 

MRI

**Figure 10:**
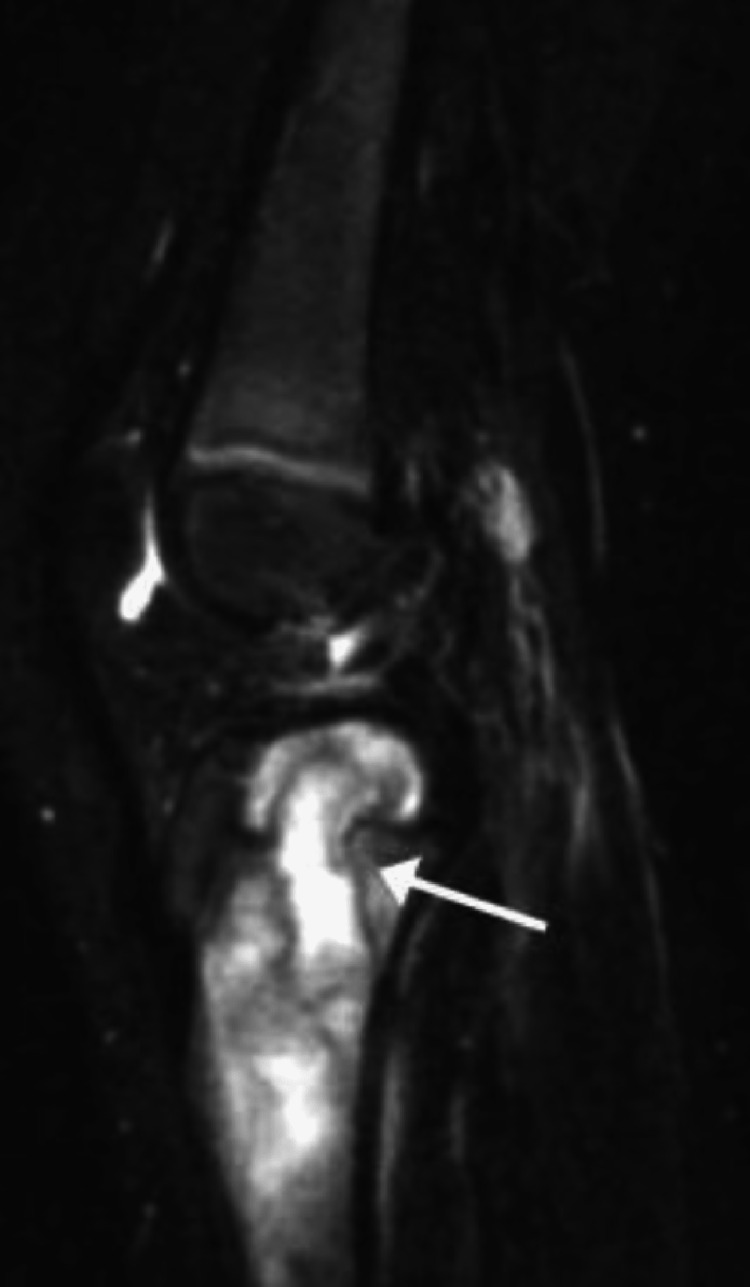
A 22-month-old girl with BCG osteomyelitis Sagittal STIR image shows diffuse bone marrow edema from the proximal diaphysis to the epiphysis, associated with the mildly increased signal intensity of the surrounding soft tissue. There is abscess formation from the metaphysis beyond the growth plate to the epiphysis with a circumferential low signal rim [17].

**Figure 11:**
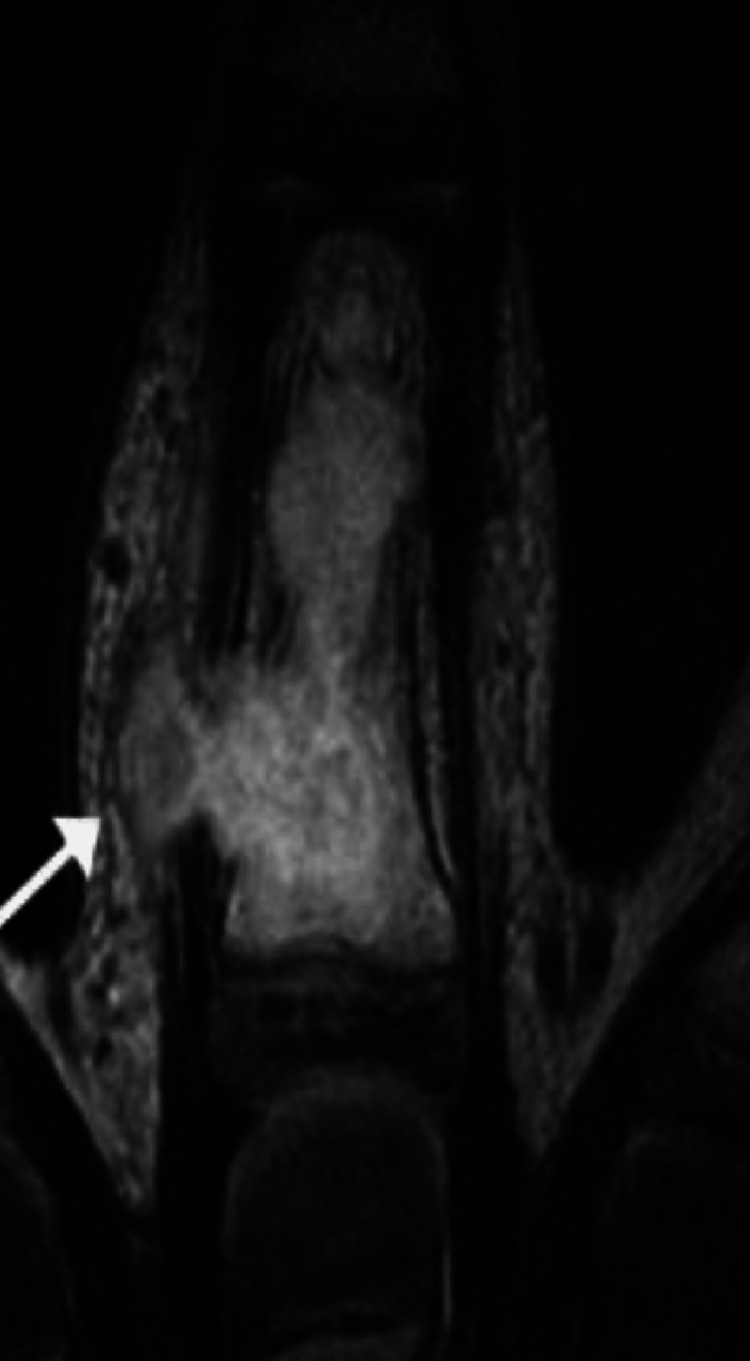
A 17-month-old girl diagnosed with BCG osteomyelitis of the phalanx The coronal T2-weighted image of the right hand shows the abscess extending into the subcutaneous tissue disrupting the cortex [17].

**Figure 12:**
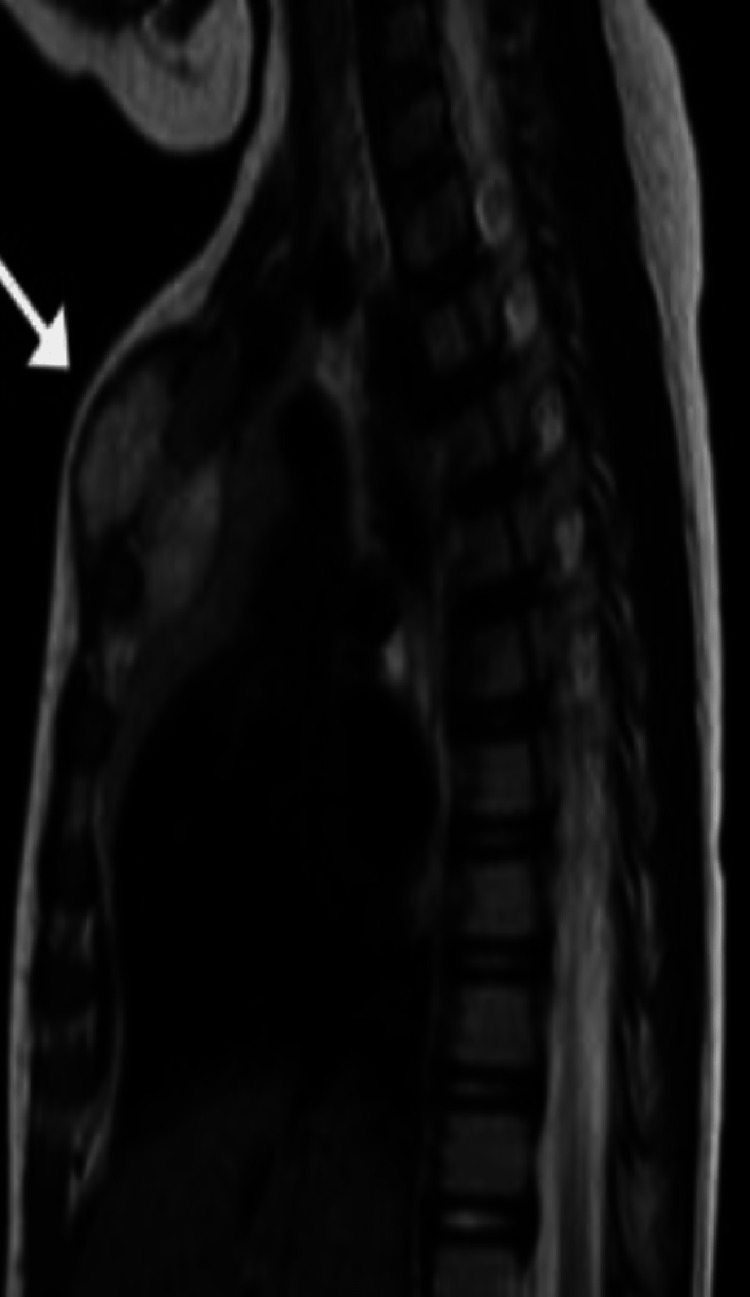
A 15-month-old girl with BCG osteomyelitis The sagittal T2-weighted image shows an encapsulated irregularly shaped abscess, spreading from inside the sternum to both ventral and dorsal surrounding soft tissue [17].

MRI is the most accurate imaging test for assessing suspected osteomyelitis, with meta-analysis showing a sensitivity of 90% and specificity of 79% [23]. According to an MRI imaging study by Tsujioka et al. [17], patients with BCG osteomyelitis were more likely to develop abscesses; intraosseous abscesses tended to be larger, and abscess capsules tended to be thicker than those in patients with pyogenic osteomyelitis (Figures 10-12).

**Figure 13:**
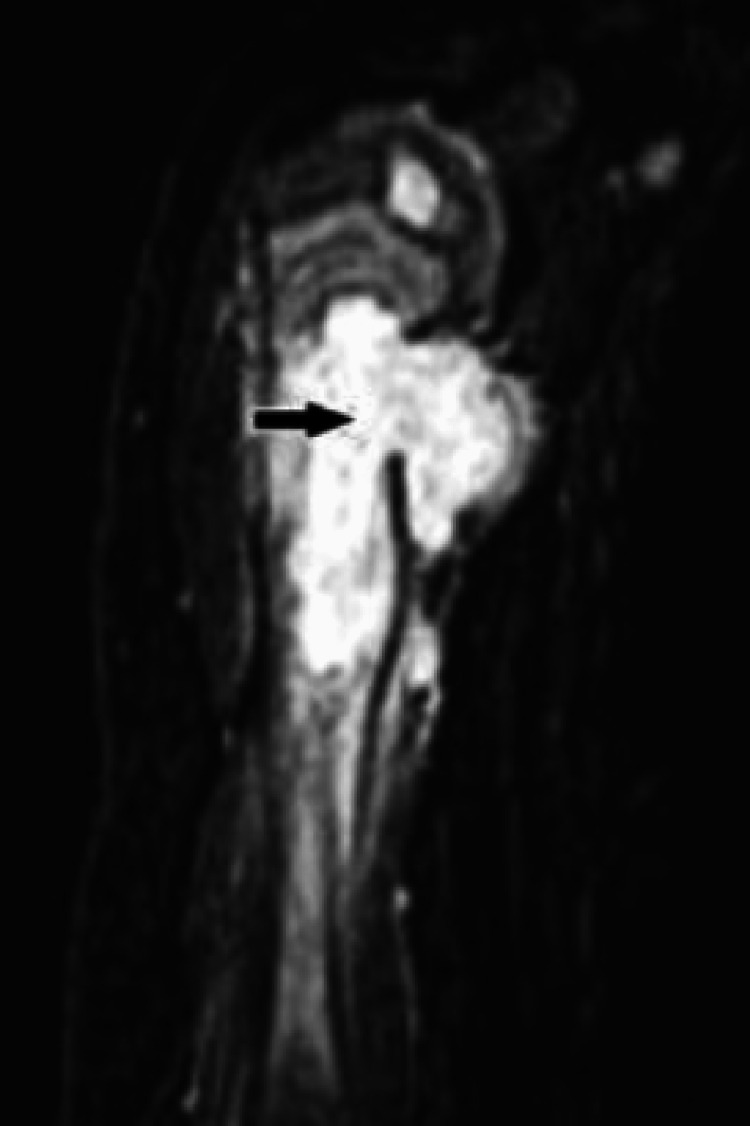
MRI showing a destructive lesion in the upper metaphysis of the right humerus

**Figure 14:**
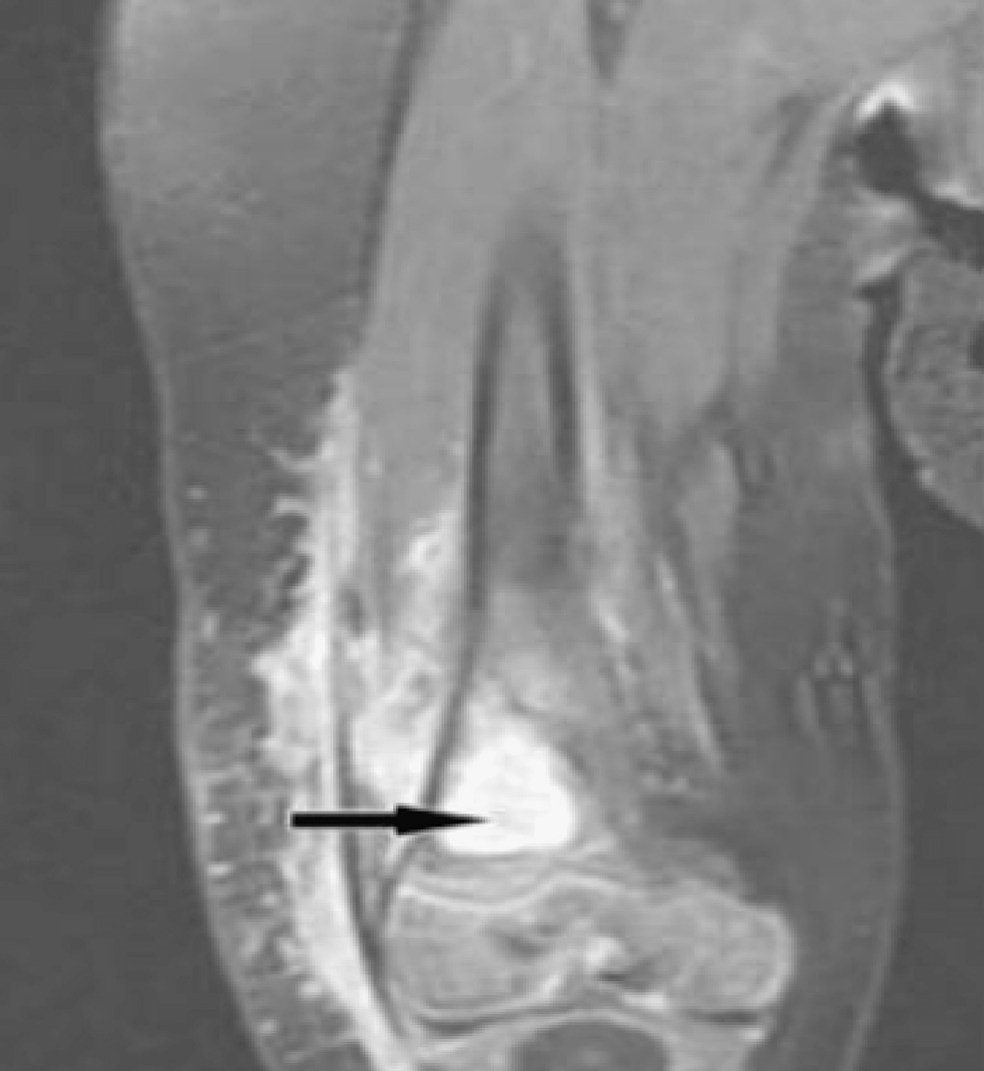
MRI showing a lytic lesion in the distal right femur with soft tissue extension

**Figure 15:**
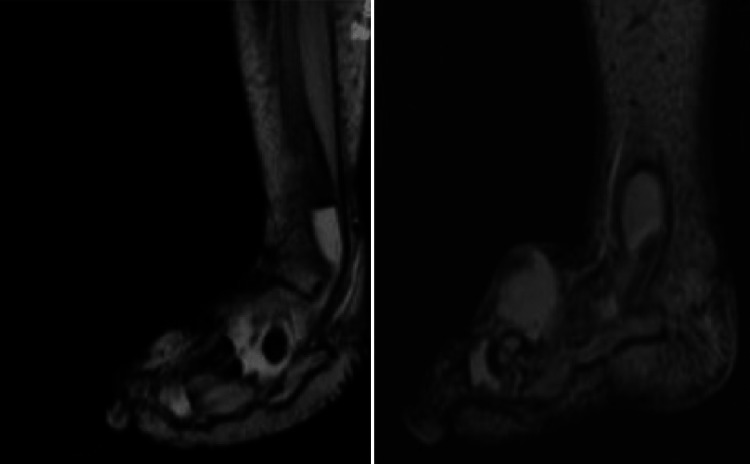
MRI showing the intramedullary expansion of the 1st metatarsal bone with cortical thinning and the presence of periosteal reaction Soft tissue around the metatarsal showed some signal changes with extension dorsomedially that caused bulging [14].

In Saudi Arabia, Al-Jassir et al. [14] reported that their patients' MRI showed a lytic lesion with cortical disturbance (Figures 13, 14). Alzomor et al. [16] reported a patient who had an intramedullary extension of the first metatarsal bone with cortical diminishing and the presence of periosteal reaction; the surrounding soft tissue showed some signal changes with dorsomedial extension causing bulging (Figure 15).

Management

Medical Treatment

No treatment guidelines are available for BCG-related osteomyelitis in children with competent immune systems. Using an antituberculous regimen, either first-line treatment with rifampicin, isoniazid, and ethambutol or second-line treatment with levofloxacin or macrolides showed a relatively good response in BCG-induced complications, according to patient status, site, or a number of lesions. Additionally, limited data are available on the antimicrobial susceptibility of BCG strain-induced osteomyelitis. Ritz et al. [3] studied the susceptibility of different BCG strains to antimicrobial regimens. They found that all BCG strains were resistant to pyrazinamide, as all the strains were derived from M. bovis attenuation, which is naturally resistant to pyrazinamide. Among the different strains used in the study by Ritz et al. [3], the Danish strain, used in Saudi Arabia, and the Connaught strain showed low-level resistance to isoniazid. However, the clinical significance of this type of resistance remains unclear. The World Health Organization reported five children diagnosed with isoniazid-resistant BCG lymphadenitis after immunization with the Danish strain; however, the strain’s known resistance to isoniazid did not justify any change in immunization policy; moreover, the concentration of isoniazid after 10 mg/kg dosage exceeded 4 µg/mL suggesting low clinical importance for resistance [24,25].

Huang et al. [21] treated their patients with a single lesion other than lesions of the vertebrae with isoniazid or rifampicin for six to nine months. However, patients with a vertebral lesion or multiple lesions were associated with a high failure rate if treated with the first-line antituberculous medications (isoniazid, rifampicin, pyrazinamide, and ethambutol); however, they showed a good response and rapid recovery using second-line regimens, such as levofloxacin or macrolides. These findings were also supported by previous studies [26-28].

Hesseling et al. [29] suggested a four-drug treatment plan that lasts for nine months if the goal is to slow the progression of the disease and achieve the best possible outcome. Lin et al. [30] found that the medical regimen usually had a good effect on BCG osteomyelitis and proposed that a 6-12-month course of isoniazid and rifampicin be used as an initial treatment for the condition. Isoniazid was used for an extended period in Finland, where the Copenhagen strain was responsible for 40% of all BCG infections. During this time, three-drug regimens that included streptomycin were favored in Finland and Sweden [9,31].

Surgery

Surgery may help in the treatment of BCG osteomyelitis; however, there is a risk that the removal of bone lesions may worsen the condition. Patients with damaged weight-bearing joints, growth plates, or vertebrae are at greater risk of complications. Patients who underwent diagnostic procedures merely avoided significant complications; in contrast, those who underwent major surgical approaches did not benefit in the short term, according to the findings of Lin et al. [30]. As a result of the fact that patients who presented with more severe symptoms were more likely to undergo surgery, we advise patients who have BCG osteomyelitis to undergo as few surgical procedures as is practically possible. Although it had long been considered that the spine is not implicated in BCG-related osteomyelitis, seven cases of BCG-related osteomyelitis involving the spine have been reported, including one with multifocal lesions. In such cases, major surgical approaches are dangerous, and their risk outweighs the benefits [30]. The treatment of BCG osteomyelitis, especially in immunocompetent children, is highly subjective, as the treatment options may differ according to the number and site of the lesions. In addition, patients only undergoing diagnostic surgical procedures instead of major surgical interventions may avoid major complications.

Management in Saudi Arabia

There are no national guidelines to manage BCG-induced osteomyelitis; therefore, we have described the different approaches used in Saudi Arabia to manage these cases. Al-Jassir et al. [14] performed surgical irrigation, thorough curettage, and debridement, and the patient underwent the entire course of anti-TB medication administered by the Infectious Diseases Division of their Institute's Infectious Diseases Unit (triple-drug treatment including rifampicin, that necrotic tissue and drain pus since there was a sinus that extended to the first metatarsal bone of the patient's foot. Initially, the patient was prescribed rifampicin, isoniazid, and pyrazinamide for two months. Streptomycin was provided for two weeks at the end of the treatment. The patient underwent frequent follow-ups at the Pediatric Infectious Disease Clinic where she exhibited complete clinical remission. Following the culture findings, treatment with pyrazinamide was discontinued, although treatment with rifampicin and isoniazid was maintained. In a study similar to this, Al-Arabi et al. [15] gave their patient with BCG osteomyelitis rifampicin, isoniazid, and ethambutol. At the completion of the nine-month checkup, the child's overall health seemed to be stable, and there was no trace of an exterior lesion where the examination was performed. According to earlier research, patients with immunocompetent immune systems who receive adequate chemotherapeutic treatment have a better chance of recovering from BCG osteomyelitis.

## Conclusions

Osteomyelitis caused by BCG vaccination is a rare adverse effect that can occur in immunocompetent children, 4-24 months after vaccination. The metaphyses or epiphyses of long bones are the most frequently affected. BCG osteomyelitis is challenging to diagnose because its symptoms are frequently nonspecific, and because of the delay between vaccination and symptom onset. MRI or CT scans typically show large, destructive interosseous abscesses. There are no current guidelines for treating osteomyelitis, but antituberculosis regimens, especially isoniazid and rifampicin, have been shown to be effective in previous literature; however, surgical intervention was not preferred for its complication, with few studies reporting a minor surgical intervention and a good outcome.

As BCG osteomyelitis is an infrequent complication, especially in immunocompetent children, its diagnosis is time-consuming. Therefore, it is critical to inform healthcare workers of this possible complication to make the diagnosis more straightforward and avoid confusion with pyogenic osteomyelitis. As only a few cases have been reported, further studies in Saudi Arabia are required for evidence-based guidelines applicable to actual practice to be established.
